# Comparison of the Vertical and the Highest Point of Shoulder Methods in Brachial Plexus Block

**Published:** 2009-03

**Authors:** Kiritoglu S, Basaranoglu G, Comlekci M, Suren M, Erkalp K, Teker G, Saidoglu L

**Affiliations:** *Department of Anasthesia, Vakif Gureba Education and Teaching Hospital, Istanbul, Turkey*

**Keywords:** brachial plexus block, the highest point of shoulder, technique, reliability, complication, infraclavicular block, pain, anesthetics, vertical

## Abstract

**Background and Aim:**

Brachial plexus block by the highest point of the shoulder method may decrease the rate of complication in comparing with the vertical method because the needle is more lateral in the former. We aimed to investigate the highest point of the shoulder block technique against the vertical infraclavicular plexus method regarding the success rates and complications.

**Patients and Methods:**

Thirty patients with ASA I-III undergoing elective surgery were included in this study. Patients were divided into two groups, randomly. Group 1 was the highest point of the shoulder method (n of 15), and goup 2 was the vertical approach technique (n of 15). The extensor motor response of hand, wrist and elbow (The target nerves in the operation area: n. medianus, n. ulnaris, n. radialis and n. musculocutanaeus) was obtained by neurostimulation technique. Then, 30 ml bupivacaine (0.5%) was used for the initial block. Spread of analgesia and sensory and motor blocks were evaluated every 5 minutes by an anesthesiologist who was blind to the block techniques.

**Statistical Analysis:**

*T-test* and *Mann-Whitney U test* were used.

**Results:**

Successful block was achieved in all patients in both groups. There was no difference among the groups for the onset of block and the duration of block (both sensory and motor), the number of attempt, and the depth of the neddle. One patient developed pneumothorax in group 2. Procedure time of the block was longer in group 2 than in group 1 (p<0.05).

**Conclusions:**

The highest point of shoulder method with a less complication rate and shorter procedure time has a comparable success rate to vertical approach technique.

## INTRODUCTION

The highest point of the shoulder method has been reported as a technique for brachial plexus block by our group (1). The main advantages of this technique are to provide easily identificable landmarks, high success and fewer complication rate. In our earlier study, we had found that this technique was successful in 93% (of the 54 patients with ASA Physical Status I–III having either orthopedic surgery of the upper limb or neurosurgery) with the 0% complication rate. Many techniques for infraclavicular brachial plexus block other than the highest point of shoulder method have been described (2-6). The success rate of these various infraclavicular brachial plexus block methods differs from 57% to 99%. In this study, we aimed to compare the highest point of shoulder method with the vertical infraclavicular block approach.

## PATIENTS AND METHODS

After institutional local medical ethical committe approval and informed consent, 30 ASA physical status I-III patients scheduled for arm and hand surgery were included in this study. Exclucion criteria were pregnancy, allergy to local anesthetics, coagulopathy, any previous neurologic damage to the brachial plexus.

Standart monitorization such as electrocardiography, pulse oximetry and noninvasive blood pressure measuring was applied to the each patient after arrival to the operating room. The patients were in supine position and arms were adducted. Hands were in resting on the abdomen during the procedure. The physician stands beside to the arm to be blocked.

**Figure 1:**
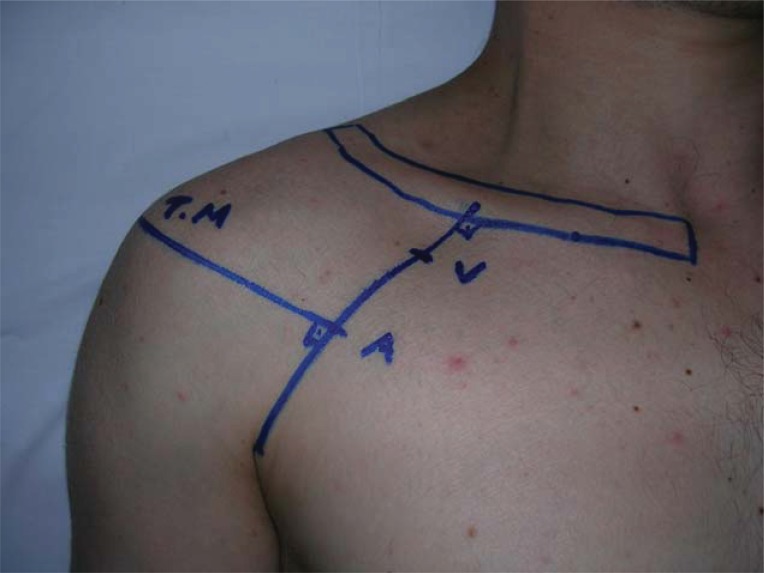
“A” indicates needle insertion area. “V”, Vertical approach; “TM”, Tuberculum majus of capitis humeri (the highest point of shoulder).

**Figure 2:**
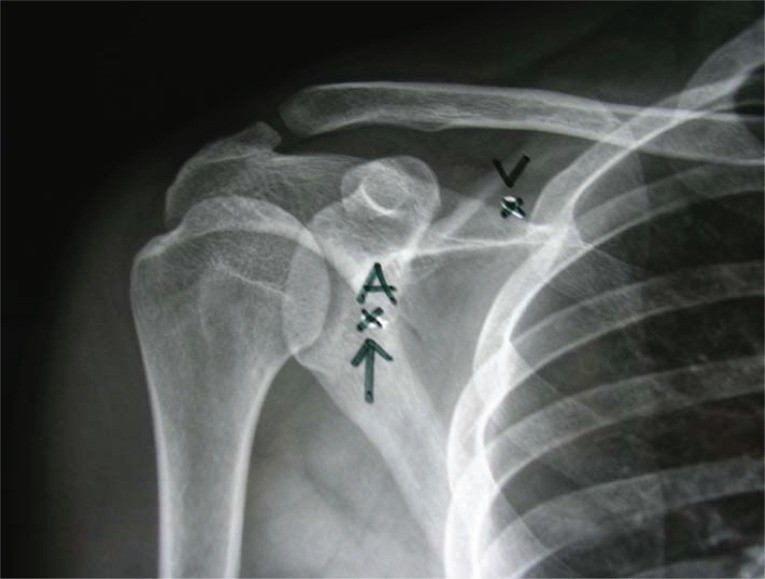
Posterior-anterior X-ray shows the distance between the neddle insertion point and the thorax wall.

In group 1, the whole length of the clavicle was marked. The axillary artery was palpated in the anterior axiller fossa. Then, a line was drawn from the artery to the midpoint of the clavicle. A line perpendiculer to this first line was then drawn from the tuberculum majus of capitis humeri which is the highest point of shoulder. The block needle was inserted at the intersection of the two lines (Figures 1 and 2). The stop clock was started to determine the time required to perform the block at that point. After a local skin infiltration with a less than 3 ml lidocaine (2%), a 100 mm 21 gauge stimulating needle (Stimplex A B. Braun Melsungen AG, Japan) connected to the nerve stimulator (Multistimplex, Pajunk, Germany). The needle was inserted to the skin, perpendicularly. The stimulation frequency was 2 Hz, duration of the stimulating pulse was 0.1 miliseconds. Initial current intensity was 1.4 mA. An upper extremity extensor motor response was obtained in the hand, wrist and elbow corresponding to median, radial and ulnar nerve stimulation. Then, the intencity of the current reduced to 0.4 mA, progressively. A negative aspiration was performed after each 5 cc of bupivacaine which was injected by the anasthesiologist. Totally, 30 ml of bupivacaine 0.5% were injected. The stop clock was stopped at that point.

In group 2, the vertical block has been performed according to the Kilka’s method (2). In this method, the patient was in supine position and the needle insertion site just under the mid-clavicle. At that point, the stopclock was started to determine time required to perform the block. The same procedure, neurostimulation preparations and the dosage of the drugs were given in both groups in the current study. Spread of analgesia to the arm was assessed every 5 minutes.

### Statistical analysis

The data were presented as mean ± SD and had been analyzed by SPSS 13 (SPSS Inc, Chicago, IL, USA). Features of groups were compared with each other by *t-test* and *Mann-Whitney U test*. Probability (p) values < 0.05 were considered as statistically significant.

## RESULTS

Demographic characteristics of the groups were presented in Table 1. Complete block has been achieved in all the patients within the two groups. As shown in Table 2, there was no difference regarding the time of the onset of block, the number of attempts, the depth of the needle between the groups. The total time to perform the block was significantly shorter in the group 1 than in the group 2, as shown in Table 3. A case with pneumothorax has been observed in group 2 (by vertical method). None of the patients had vascular puncture, Horner’s Syndrome, hemothorax or local anesthetic toxicity.

**Table 1:** Demographic characteristics

	Group 1 (n of 15)	Group 2 (n of 15)	p

Gender M/F%	60/40	67/33	NS^a^
BMI (Body Mass Index)	24.1	25.7	NS
Age (year)	36.5	47.5	NS

a non-significant.

**Table 2:** The onset of block time (OBT), time of motor block (TMB), and time of sensitive block (TSB)

Mean ± SD	Group 1 (n=15)	Group 2 (n=15)	t	p

OBT (min)	12.47 ± 1.3	12.47 ± 0.83	0.00	1.00
TMB (min)	601.33 ± 70.19	636.67 ± 26.9	1.82	0.079
TSB (min)	624.67 ± 80.79	671.33 ± 30.67	2.09	0.051

**Table 3:** The number of attempts for block, needle depth, time of block procedure

Mean ± SD	Group 1 (n=15)	Group 2 (n=15)	z	p

The number of attempts	2.47 ± 0.83	2.47 ± 0.64	0.272	0.785
Needle depth (cm)	5.13 ± 0.83	5 ± 0.53	0.362	0.717
Block procedure time (min)	4 ± 0.76	5.2 ± 1.15	2.882	0.004^a^

a non-significant.

## DISCUSSION

Technically, the highest point of shoulder method provided a shorter procedure time for the brachial plexus blockade than the vertical method. We did not find any major difference between the techniques other than the procedure time. Otherwise, both techniques provided a good anasthesia for the infraclavicular plexus block for the wrist, hand, forarm surgeries. Although reported success rates of various infraclavicular brachial plexus block methods differs from 57% to 99% in the current literature (7-12), both techniques was performed with 100% success rate in our study.

Excessive plasma levels of bupivacaine may cause systemic reactions involving the central nervous system and the cardiovascular system. The central nervous system effects are characterized by excitation or depression. The first manifestation may be nervousness, dizziness, blurred vision, or tremors, followed by drowsiness, convulsions, unconsciousness, and possibly respiratory arrest. Other central nervous system effects may be nausea, vomiting, chills, constriction of the pupils, or tinnitus (13).

Main considerations about the techniques of infraclavicular brachial plexus block are the rate of complications, the reliability of the technique, success rate of the technique, and to finding landmarks easily, easy applications and easy approach methods (7-12). In the literature, the vertical method usually compared with axillar method (2-6). Pneumothorax risk in vertical approach is high due to the technique. In fact, Kilka *et al*. who developed vertical technique did not report any pneumothorax in their series (2). Others reported the prevalence of pneumothorax from 0.2 to 0.7%. Kilka *et al*. also reported the rate of venoupuncture as 10%. In our study, pneumothorax and venoupuncture were not seen in both group.

In conclusion, the highest point of shoulder method with a less complication rate and shorter procedure time has a comparable success rate to over vertical approach technique.

## References

[1] Comlekci M, Basaranoglu G, Suren M, Bay B (2006). An approach to infraclavicular brachial plexus block: the highest point of the shoulder as a reference. Anesth. Analg.

[2] Kilka HG, Geiger P, Mehrkens HH (1995). Infraclavicular vertical brachial plexus blockade. A new method for anesthesia of the upper extremity. An anatomical and clinical study. Anaesthesist.

[3] Rettig HC, Gielen MJ, Boersma E, Klein J (2005). A comparison of the vertical infraclavicular and axillary approaches for brachial plexus anaesthesia. Acta. Anaesthesiol. Scand.

[4] Neuburger M, Kaiser H, Ass B, Franke C (2003). Vertical infraclavicular blockade of the brachial plexus (VIP). A modified method to verify the puncture point under consideration of the risk of pneumothorax. Anaesthesist.

[5] Heid FM, Jage J, Guth M, Bauwe N (2005). Efficacy of vertical infraclavicular plexus block vs. modified axillary plexus block: a prospective, randomized, observer-blinded study. Acta. Anaesthesiol. Scand.

[6] Schüpfer GK, Jöhr M (1997). Infraclavicular vertical plexus blockade: a safe alternative to the axillary approach?. Anesth. Analg.

[7] Desroches J (2003). The infraclavicular brachial plexus block by the coracoid approach is clinically effective: an observational study of 150 patients. Can. J. Anaesth.

[8] Borgeat A, Ekatodramis G, Dumont C (2001). An evaluation of the infraclavicular block via a modified approach of the Raj technique. Anesth. Analg.

[9] Kapral S, Jandrasits O, Schabernig C, Likar R (1999). Lateral infraclavicular plexus block vs. axillary block for hand and forearm surgery. Acta. Anaesthesiol. Scand.

[10] Koscielniak-Nielsen ZJ, Rasmussen H, Hesselbjerg L, Nielsen TP (2005). Infraclavicular block causes less discomfort than axillary block in ambulatory patients. Acta. Anaesthesiol. Scand.

[11] Deleuze A, Gentili ME, Marret E, Lamonerie L (2003). A comparison of a single-stimulation lateral infraclavicular plexus block with a triple-stimulation axillary block. Reg. Anesth. Pain Med.

[12] Jandard C, Gentili ME, Girard F, Ecoffey C (2002). Infraclavicular block with lateral approach and nerve stimulation: extent of anesthesia and adverse effects. Reg. Anesth. Pain Med.

[13] Side effects www.rxlist.com/marcaine-drug.htm..

